# Complete genome sequence of *Peribacillus frigoritolerans* NBTC-101, isolated from melon rhizosphere soil

**DOI:** 10.1128/mra.00937-25

**Published:** 2025-12-29

**Authors:** Wei Chen, Wenrui Liu, Yong Min, Ben Rao, Yuxi Tian, Fei Li, Qian Deng, Yueying Wang, Xiaoyan Liu, Lei Zhu, Ling Chen

**Affiliations:** 1 National Biopesticide Engineering Technology Research Center, Hubei Biopesticide Engineering Research Center, Hubei Academy of Agricultural Sciences, Biopesticide Branch of Hubei Innovation Centre of Agricultural Science and Technology 117996, Wuhan, Hubei, People's Republic of China; 2 College of Life Science and Technology, Tarim University 12483, Aral, Xinjiang, People's Republic of China; University of Wisconsin-Madison, Madison, Wisconsin, USA

**Keywords:** *Peribacillus frigoritolerans*, genome sequence

## Abstract

*Peribacillus frigoritolerans* is a widely distributed gram-positive bacterium that exhibits several notable characteristics. Here, we report the complete genome sequence of the *P. frigoritolerans* strain NBTC-101, which was isolated from the rhizosphere soil of melons. This genome sequence will help elucidate the ecological role of *P. frigoritolerans*.

## ANNOUNCEMENT

*Peribacillus frigoritolerans* is a rod-shaped, gram-positive bacterium that was initially classified as *Brevibacterium frigoritolerans* (1). *P. frigoritolerans* is widely distributed and demonstrates a high tolerance to extreme environments, as evidenced by isolates obtained from arid soils, arctic snow, food products, animals, and plants (2–4). Strains of *P. frigoritolerans* are currently documented to exhibit several notable characteristics, including phosphate neutralization activity, insect pathogenicity, and plant growth-promoting properties. In this study, we present the genome sequence of *P. frigoritolerans* strain NBTC-101, which was isolated from the rhizosphere soil of melon plants. The data will help elucidate the ecological role of *P. frigoritolerans*.

Strain NBTC-101 was isolated from the rhizosphere soil of melons collected in a greenhouse located in Xiantao City (30.20° N, 114.27° E), China. The melons had been damaged by spider mites. Soil samples were suspended in sterile water, and after thorough shaking, the supernatant was diluted in a series of gradients. From each dilution, 100 μL of the liquid was transferred and spread onto a Luria-Bertani (LB) agar plate. The plates were then incubated at 30°C overnight. Based on the varying colony morphologies, a single yellow colony with irregular edges and a smooth surface was selected for purification and further cultivation. Strain NBTC-101 was subsequently cultured in LB broth at 30°C with shaking at 220 rpm overnight for genome sequencing.

The genomic DNA of NBTC-101 was extracted using the Blood & Cell Culture DNA kits (Qiagen, Germany) following the manufacturer’s instructions. For short-read sequencing, the genomic DNA was fragmented using an LE220 focused ultrasonicator (Covaris, USA). Subsequently, DNA fragments with an average size ranging from 200 to 400 bp were selected utilizing AMPure XP magnetic beads (Beckman, Germany). A paired-end short-read sequencing library was prepared with the MGIEasy FS PCR-free library preparation set (MGI Tech Co., Ltd.). Sequencing was conducted on a DNBSEQ-T7 instrument (MGI, China) using 150-nucleotide long reads. Raw reads were processed by trimming low-quality reads and adapters with the FASTQ preprocessing program fastp v.0.23.2 (5), employing default settings. The resulting short-read data set comprised 7,062,348 reads, totaling 1,054,126,002 bp in length. For long-read sequencing, a DNA library was constructed using the SQK-LSK110 library preparation kit (Oxford Nanopore Technologies, UK). The library was sequenced with a PromethION sequencer (ONT) utilizing an R9.4.1 flow cell (FLO-MIN106). Subsequently, base calling, barcode segmentation, and adapter sequence removal from the raw sequences were performed using Guppy v.5.0.16 with default settings (6). The filtered high-quality reads totaled 173,149, with an *N*_50_ value of 19,803 bp. The two sequencing data sets were assembled into a complete circular chromosome using Unicycler v.0.5.0 (7). The genome was annotated using NCBI PGAP v.6.1 (8). All software used the default parameters. The genome features of NBTC-101 are presented in Table 1.

**TABLE 1:** Genome features of *Peribacillus frigoritolerans* NBTC-101

Features	Value
Genome size	5,515,556 bp
Genome coverage	504×
GC content	40.5%
Number of coding sequences	5,332
Number of tRNA genes	83
Number of rRNA genes	42

Utilizing an ANI calculator (9), the genome of NBTC-101 exhibits an average nucleotide identity of 99.96% with the *Peribacillus frigoritolerans* type strain DSM 8801 (GenBank accession: GCA_024169475.1). The colony morphology of NBTC-101 also conformed to the characteristics of the *Peribacillus* strain. Consequently, NBTC-101 was identified as a strain of *P. frigoritolerans*.

## Data Availability

The genome sequence of *Peribacillus frigoritolerans* strain NBTC-101 has been deposited in the GenBank database under accession number CP197091 (BioProject number: PRJNA1293079). Sequencing reads have been submitted to the SRA database under the accession numbers SRR34579682 and SRR34579683.
